# Confounding is not the only bias influencing associations of adiposity with cardiovascular disease

**DOI:** 10.1093/eurheartj/ehy133

**Published:** 2018-03-16

**Authors:** Natalie Staplin

**Affiliations:** Medical Research Council Population Health Research Unit, Clinical Trial Service Unit and Epidemiological Studies Unit, Nuffield Department of Population Health, Oxford, UK

**This editorial refers to ‘The impact of confounding on the associations of different adiposity measures with the incidence of cardiovascular disease: a cohort study of 296 535 adults of white European descent’^†^, by S. Iliodromiti *et al.*, on page 1514.**

There has been much discussion in the epidemiological literature about the so-called ‘obesity paradox’, where being overweight or obese appears to be protective for mortality in studies including individuals with prior cardiovascular disease (CVD).^1^ These inferences are based on the J-shaped associations observed between body mass index (BMI) and mortality in such populations, but it has been argued that these are a result of biases (such as selection bias^2^) rather than obesity conferring a true survival advantage. Indeed, several studies have shown that, with appropriate adjustment for confounding and reverse causality (i.e. restricting analyses to non-smokers with no prior disease and excluding the first 5 years of follow-up), overweight and obesity are associated with an increase in the risk of all-cause mortality compared with normal weight.^3^^,^^4^

In this issue of the journal, Iliodromiti and colleagues^5^ report similar findings for the association between BMI and incident CVD in the UK Biobank. After excluding participants with prior co-morbidities, the observed J-shaped associations had almost disappeared, with monotonic relationships being observed above the reference value of 22 kg/m^2^. Interestingly, the associations between the other adiposity measures considered [waist circumference (WC), waist-hip-ratio, waist to height ratio, and body fat percentage] and incidence of CVD did not appear to be influenced by confounding or reverse causality. The associations were unchanged by the exclusion of smokers and individuals with prior co-morbidities, as well as by omitting the first 2 years of follow-up. This could be a result of BMI being a poor discriminator of body fat and lean mass. Chronic illness can lead to a reduction in lean mass and subsequent weight loss,^6^ which would have a larger impact on BMI than other adiposity measures. 

The associations between BMI (restricted to participants with a BMI ≥22 kg/m^2^), WC, and waist-hip-ratio and incidence of CVD reported by Iliodromiti and colleagues^5^ are weaker than those observed in previous analyses.^7^ The Emerging Risk Factors Collaboration (ERFC) estimate that 4.56 kg/m^2^ higher BMI is associated with a 23% increased risk of CVD, defined as a composite of coronary heart disease and ischaemic stroke [hazard ratio (HR) 1.23, 95% confidence interval (CI) 1.17–1.29]. They also estimate that 12.6 cm higher WC and 0.083 higher waist-hip-ratio are associated with increases in CVD risk of 27% and 25%, respectively (HR 1.27, 95% CI 1.20–1.33; and HR 1.25, 95% CI 1.19–1.31). There are several possible explanations for these discrepancies, including: (i) differences in covariate adjustment; (ii) lack of specificity in CVD outcome definition; and (iii) measurement error.

The ERFC presented HRs with minimal adjustment for age, sex, and smoking status, so they may still have been subject to some residual confounding. Iliodromiti and colleagues^5^ additionally adjusted the associations between adiposity measures and CVD for socio-economic status and physical activity, both of which are potential confounders. Blood pressure and diabetes were also included in the models despite being likely to lie on the causal pathway between adiposity and CVD, so the HRs may have been overadjusted. However, the HRs were only marginally higher in sensitivity analyses adjusting for age, socio-economic status, smoking, alcohol, and physical activity, so adjustment for blood pressure and diabetes had little effect on the observed associations.

A broad definition of any incident event with an ICD code in the range I00–I99 is used as the primary CVD outcome by Iliodromiti and colleagues.^5^ This includes a lot of different types of cardiovascular disease, each of which might have different causal mechanisms. Studies looking at the association between adiposity measures and cardiometabolic traits suggest that adiposity affects CVD risk through lipids, glucose, and blood pressure.^8^ Therefore, we might expect associations with CHD and ischaemic stroke, but not necessarily other types of cardiovascular disease, which might weaken the observed associations. However, using a composite outcome of ischaemic heart disease and any cerebrovascular disease did not materially change the results.

**Figure 1:**
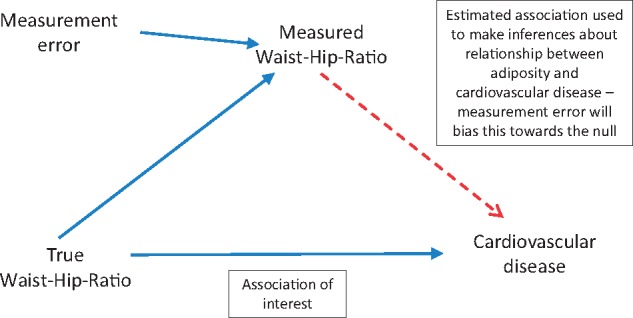
Diagram illustrating bias in the association between waist-hip-ratio and cardiovascular disease arising from measurement error. Compared with BMI and WC, waist-hip-ratio exhibits the most within-person variability and therefore is most susceptible to regression dilution bias.

Iliodromiti and colleagues^5^ acknowledge that central adiposity measures are less reproducible than BMI (i.e. there is more error in measurements of WC and associated measures such as waist-hip-ratio), but do not take these differing levels of measurement error into account in the analyses. In epidemiological analyses where the exposure is measured imprecisely, the association between the risk factor and the outcome of interest will be underestimated (*Figure 1*). This is often referred to as ‘regression dilution bias’ and can be corrected by multiplying the regression coefficient from analyses using baseline levels of the risk factor by 1/R, where R is the regression dilution ratio (which can be estimated by the correlation between pairs of measurements some time apart).^9^^,^^10^ The *P*-values associated with the HRs would remain unchanged by this correction.

Using data from the 20 000 UK Biobank participants who attended the first repeat assessment visit, regression dilution ratios for BMI, WC, and waist-hip-ratio are estimated to be 0.94, 0.83, and 0.64, respectively, for men and 0.94, 0.86, and 0.64, respectively, for women. Applying these to the HRs that have not been adjusted for the intermediate risk factors of diabetes and blood pressure (i.e. the analyses most comparable with those conducted by the ERFC), the HRs for BMI remain roughly the same (uncorrected HRs 1.16, 95% CI 1.13–1.19 for men; and 1.15, 95% CI 1.12–1.19 for women vs. corrected HRs 1.17, 95% CI 1.14–1.20 for men; and 1.16, 95% CI 1.12–1.20 for women). The strengths of the associations for WC have increased slightly (uncorrected HRs 1.13, 95% CI 1.10–1.17 for men; and 1.18, 95% CI 1.15–1.21 for women vs. corrected HRs 1.16. 95% CI 1.12–1.20 for men; and 1.21 95% CI 1.18–1.25 for women), but the associations for waist-hip-ratio have nearly doubled (uncorrected HRs 1.11, 95% CI 1.08–1.14 for men; and 1.12, 95% CI 1.09–1.14 for women vs. corrected HRs 1.18, 95% CI 1.13–1.23 for men; and 1.19, 95% CI 1.15–1.24 for women). However, the corrected associations for all three measures are still weaker than those observed in the ERFC.

The waist to height ratio was not reported in the ERFC meta-analysis as it is highly correlated with WC (*r* = 0.95).^7^ As expected, Iliodromiti and colleagues^5^ show that WC and waist to height ratio have similar strengths of association with CVD, suggesting that there is little additional value in the waist to height ratio. Body fat percentage has not been previously studied in such a large cohort, so reliable estimates of its association with CVD are not available for comparison. However, it does exhibit more within-person variability than BMI (regression dilution ratio 0.87 for both men and women) so its association with CVD should also be adjusted for regression dilution bias (uncorrected HRs 1.08, 95% CI 1.06–1.11 for men; and 1.14, 95% CI 1.10–1.17 for women vs. corrected HRs 1.09, 95% CI 1.06–1.12 for men; and 1.16, 95% CI 1.12–1.20 for women).

While associations between BMI and CVD can be biased by reverse causality if participants with prior disease are not excluded, other adiposity measures are more likely to be measured with error (particularly the waist-hip-ratio) and hence are susceptible to regression dilution bias, and so their associations with CVD may be stronger than suggested by Iliodromiti and colleagues.^5^

**Conflict of interest:** the CTSU has a staff policy of not accepting honoraria or other fees from the pharmaceutical industry, expect for the reimbursement of costs to participate in scientific meetings (www.ctsu.ox.ac.uk). N.S. reports grants from Boehringer Ingelheim, outside the submitted work.
